# Supplementary figures and images for: Human promoter directionality is determined by transcriptional initiation and the opposing activities of INTS11 and CDK9
Source: eLife. 2024 Jul 8;13:RP92764. doi: 10.7554/eLife.92764 (PMC11230626; doi:10.7554/eLife.92764)

FIGURE 1A

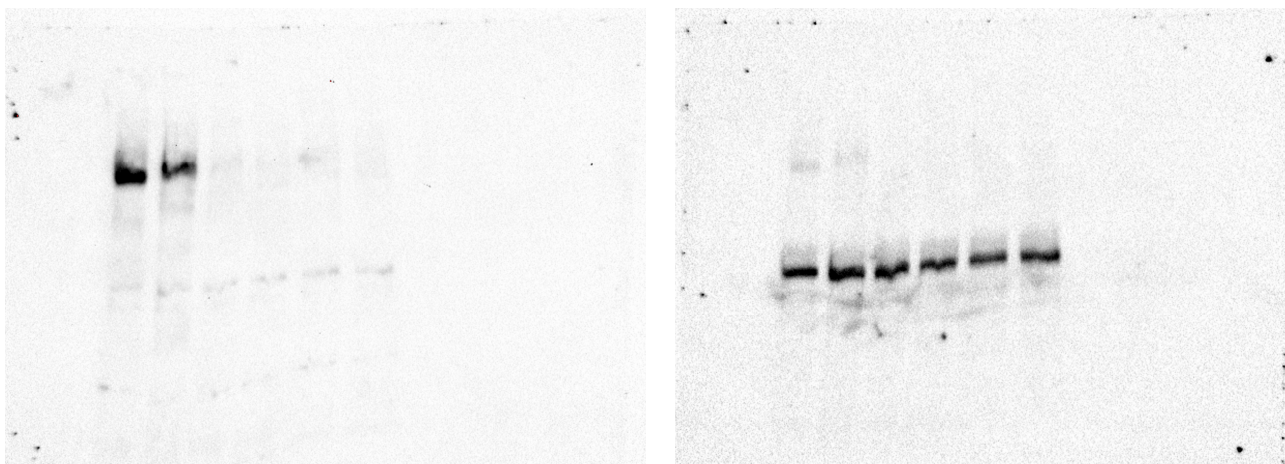

Supplement: Figure 1—source data 1. [file elife-92764-fig1-data1.pdf]

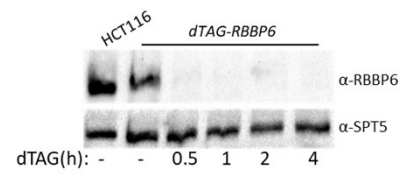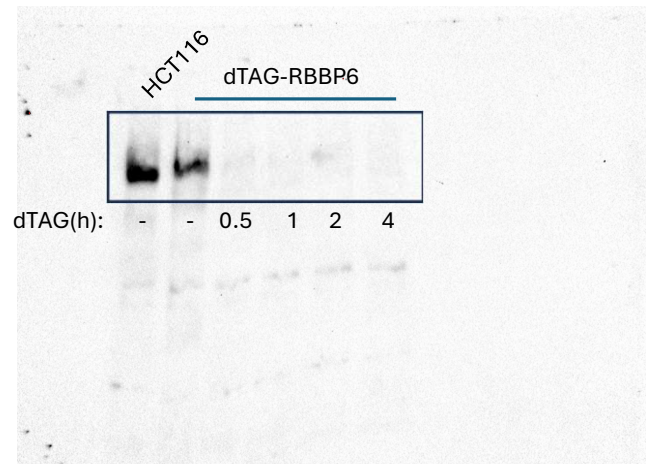

RBBP6

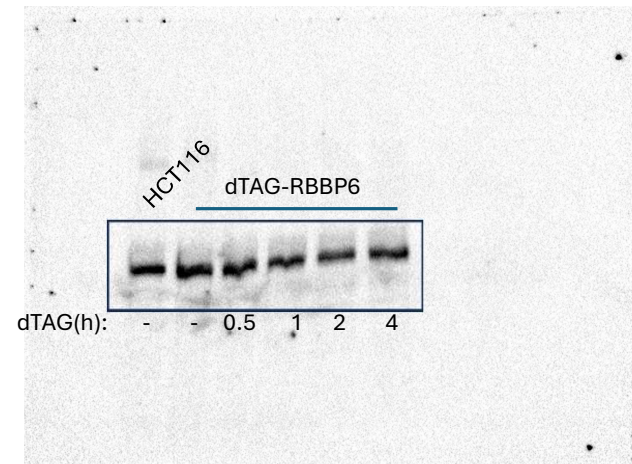

SPT5

Supplement: Figure 1—source data 2. [file elife-92764-fig1-data2.pdf]

Figure 1 – figure supplement 1

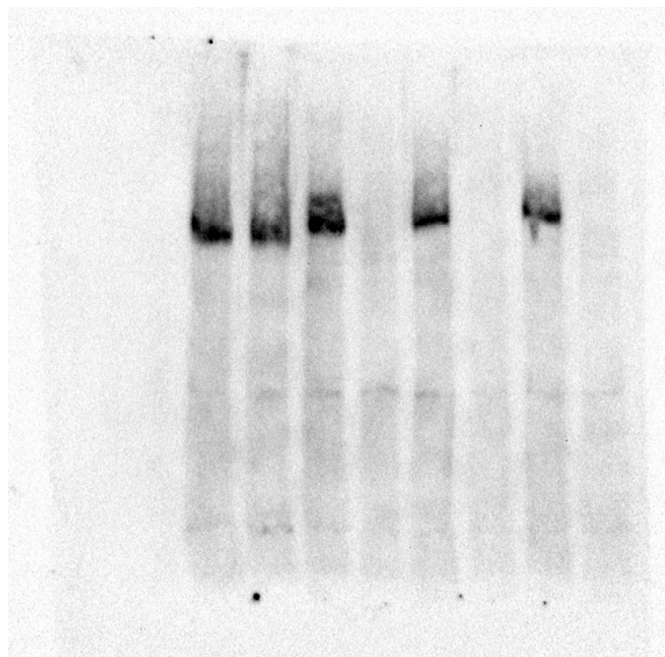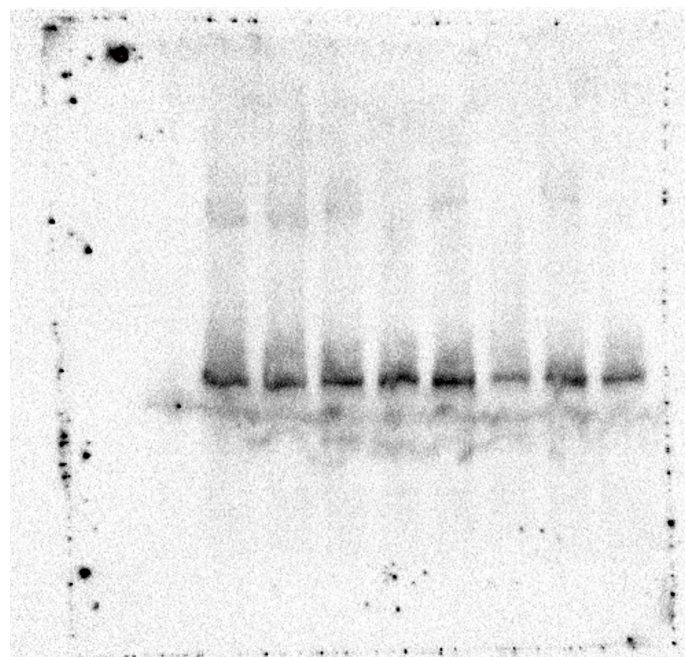

Supplement: Figure 1—figure supplement 1—source data 1. [file elife-92764-fig1-figsupp1-data1.pdf]

Figure 1 – Figure supplement 1

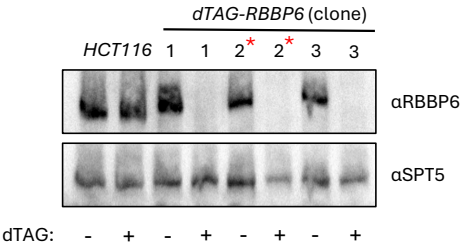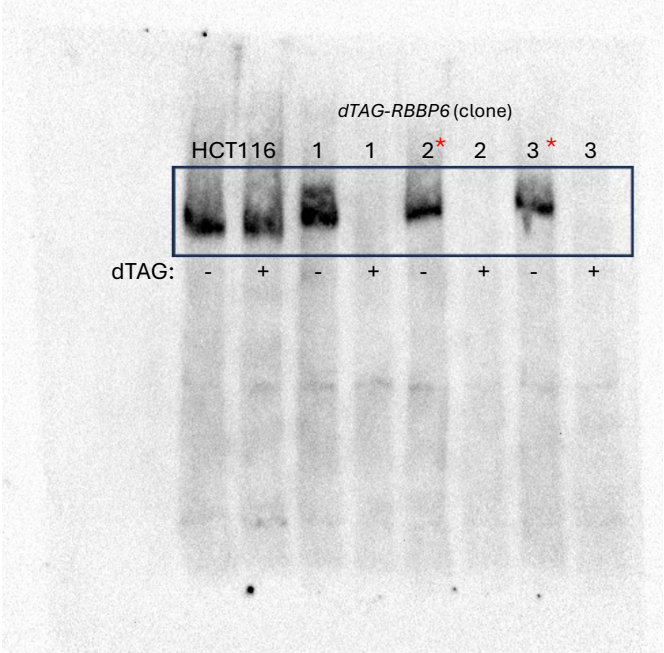

RBBP6

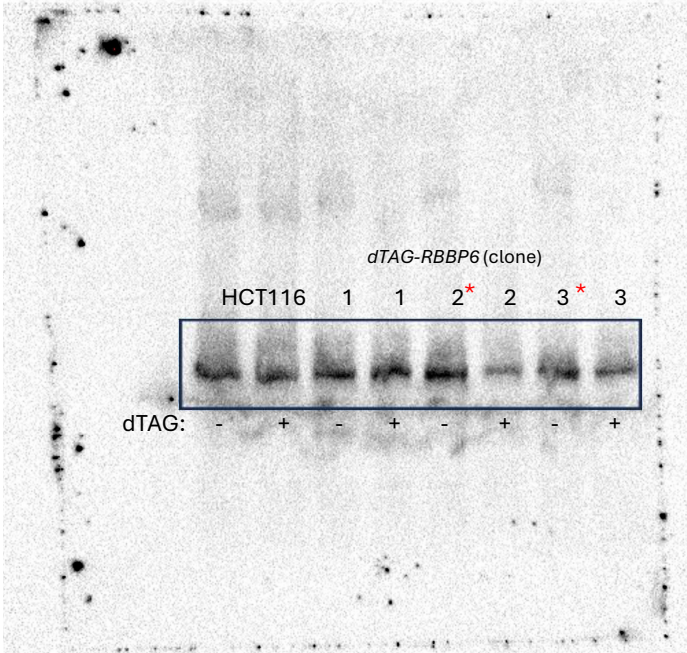

SPT5

Supplement: Figure 1—figure supplement 1—source data 2. [file elife-92764-fig1-figsupp1-data2.pdf]

FIGURE 2A

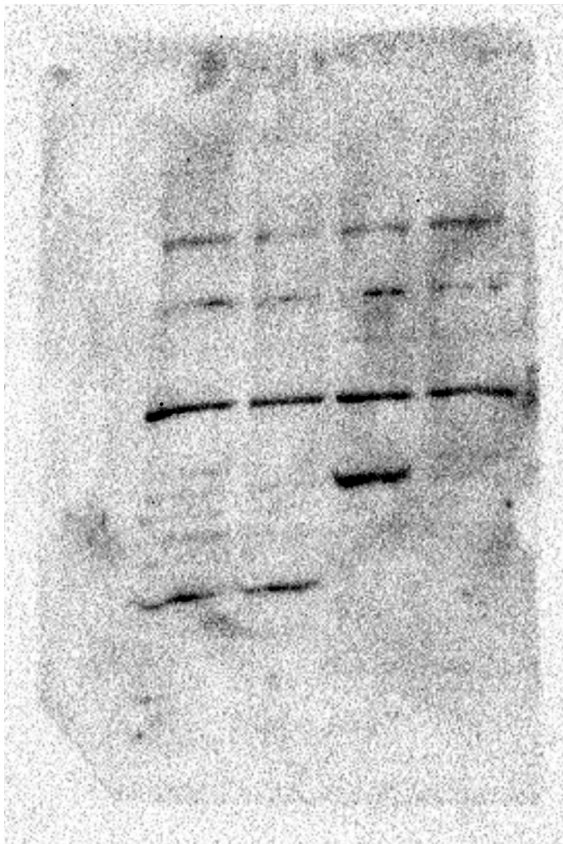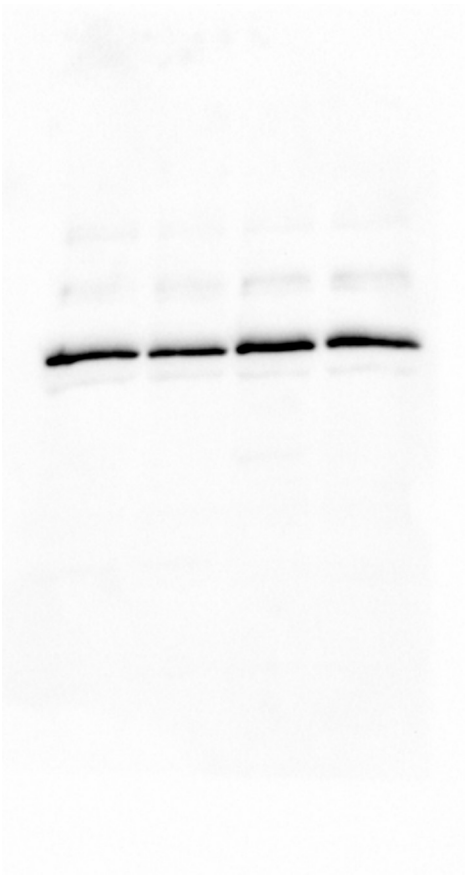

Supplement: Figure 2—source data 1. [file elife-92764-fig2-data1.pdf]

FIGURE 2A

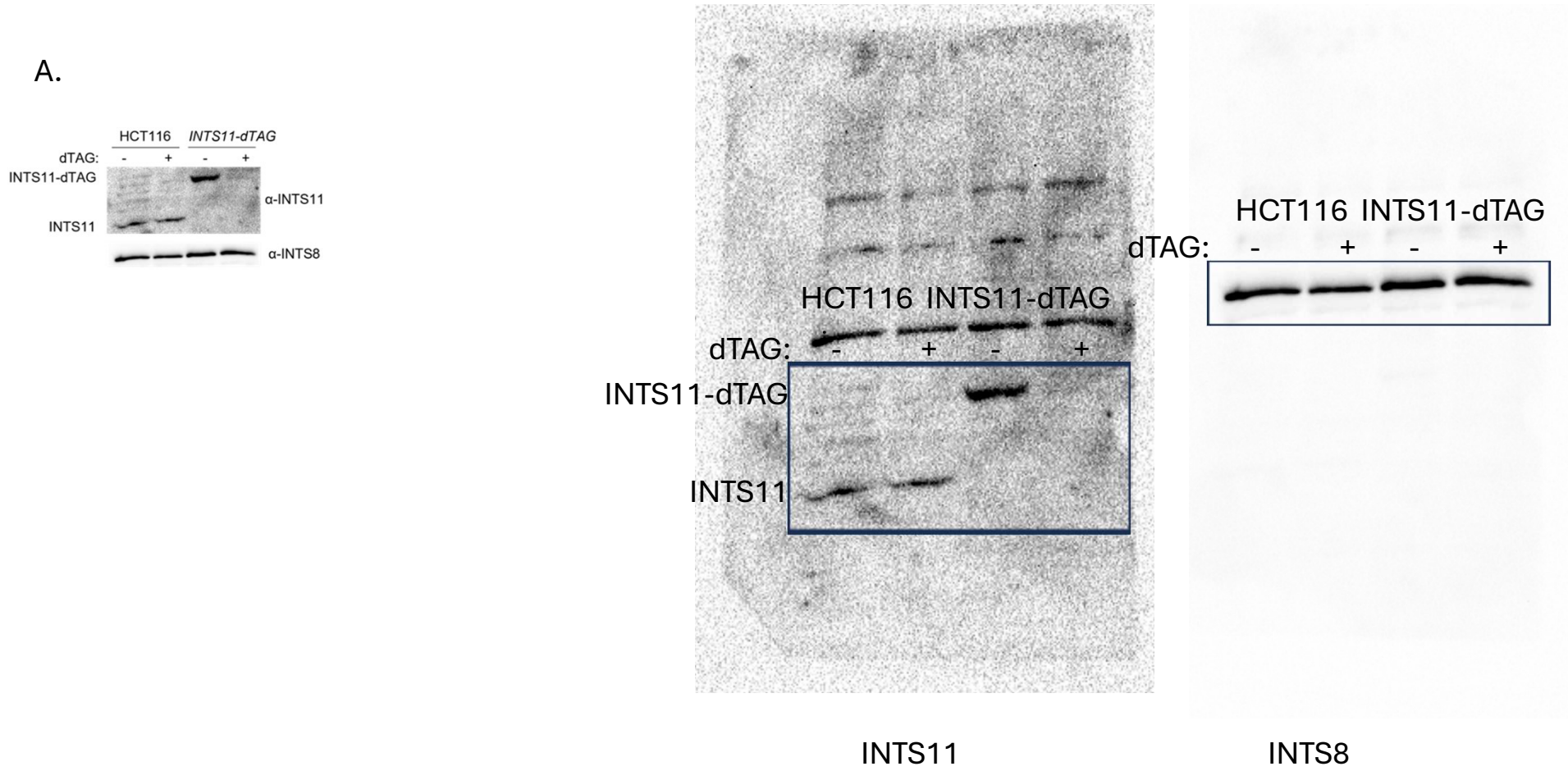

Supplement: Figure 2—source data 2. [file elife-92764-fig2-data2.pdf]

FIGURE 4E

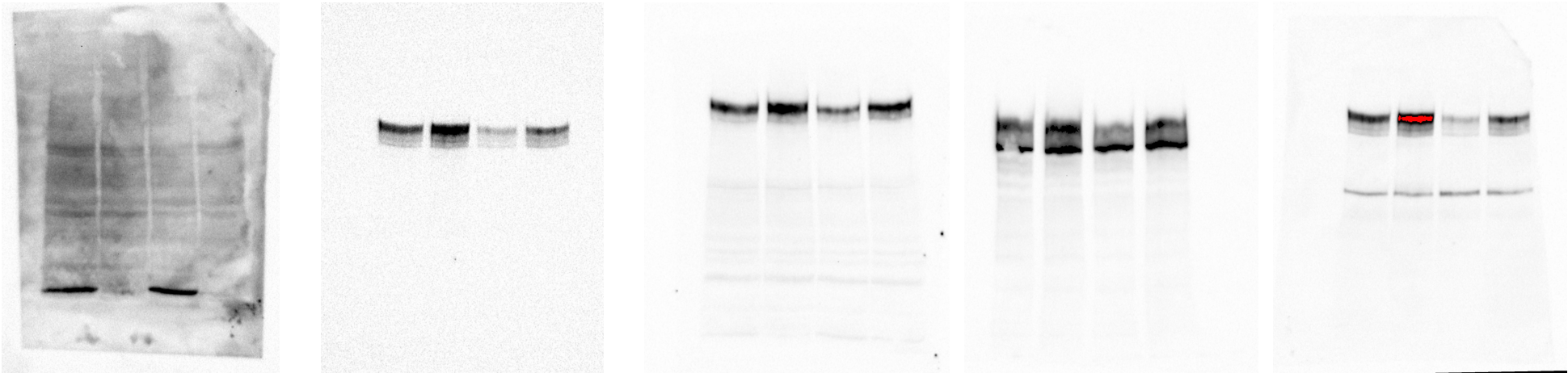

Supplement: Figure 4—source data 1. [file elife-92764-fig4-data1.pdf]

FIGURE 4E

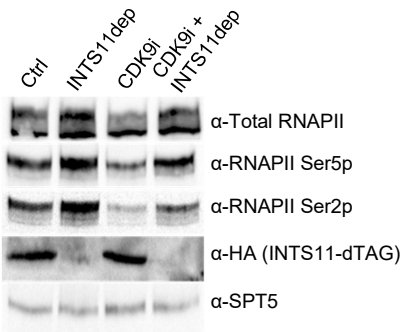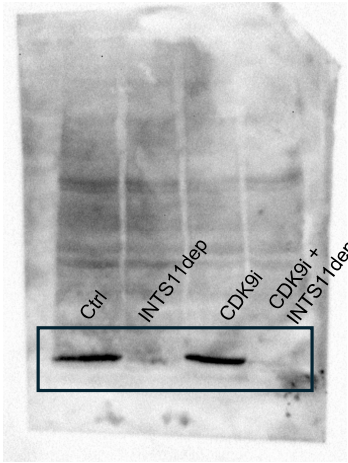

HA

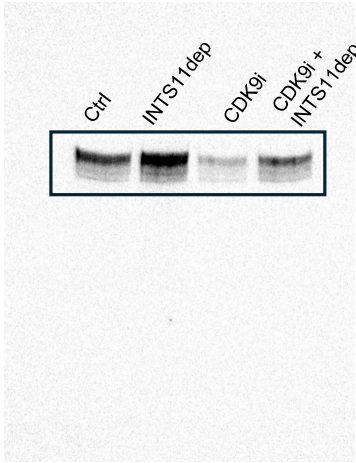

Ser2p

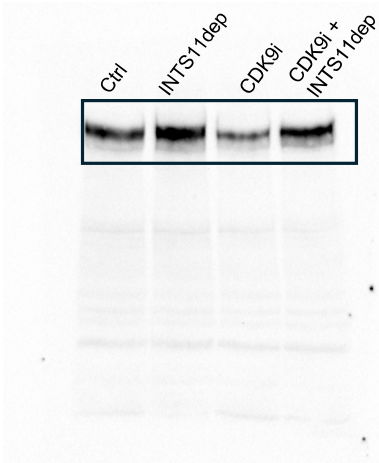

Ser5p

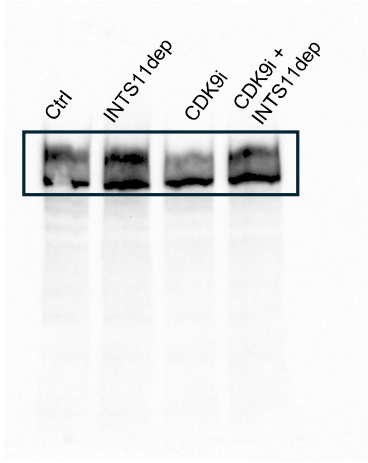

RNAPII

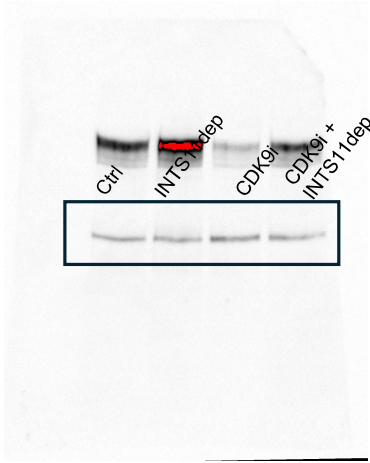

SPT5

Supplement: Figure 4—source data 2. [file elife-92764-fig4-data2.pdf]

Figure 4 – figure supplement 1 – source data 1

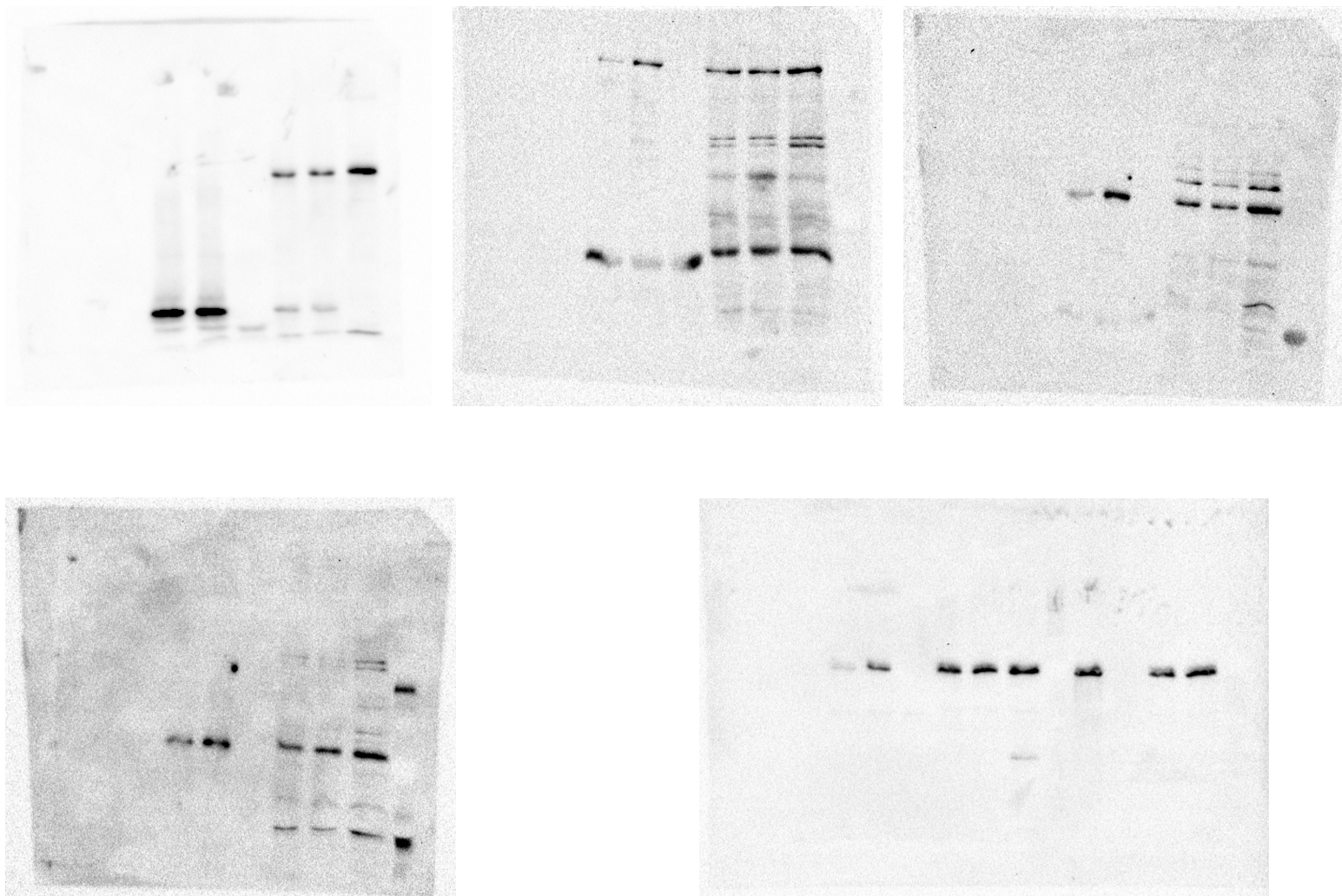

Supplement: Figure 4—figure supplement 1—source data 1. [file elife-92764-fig4-figsupp1-data1.pdf]

Figure 4 – figure supplement 1 – source data

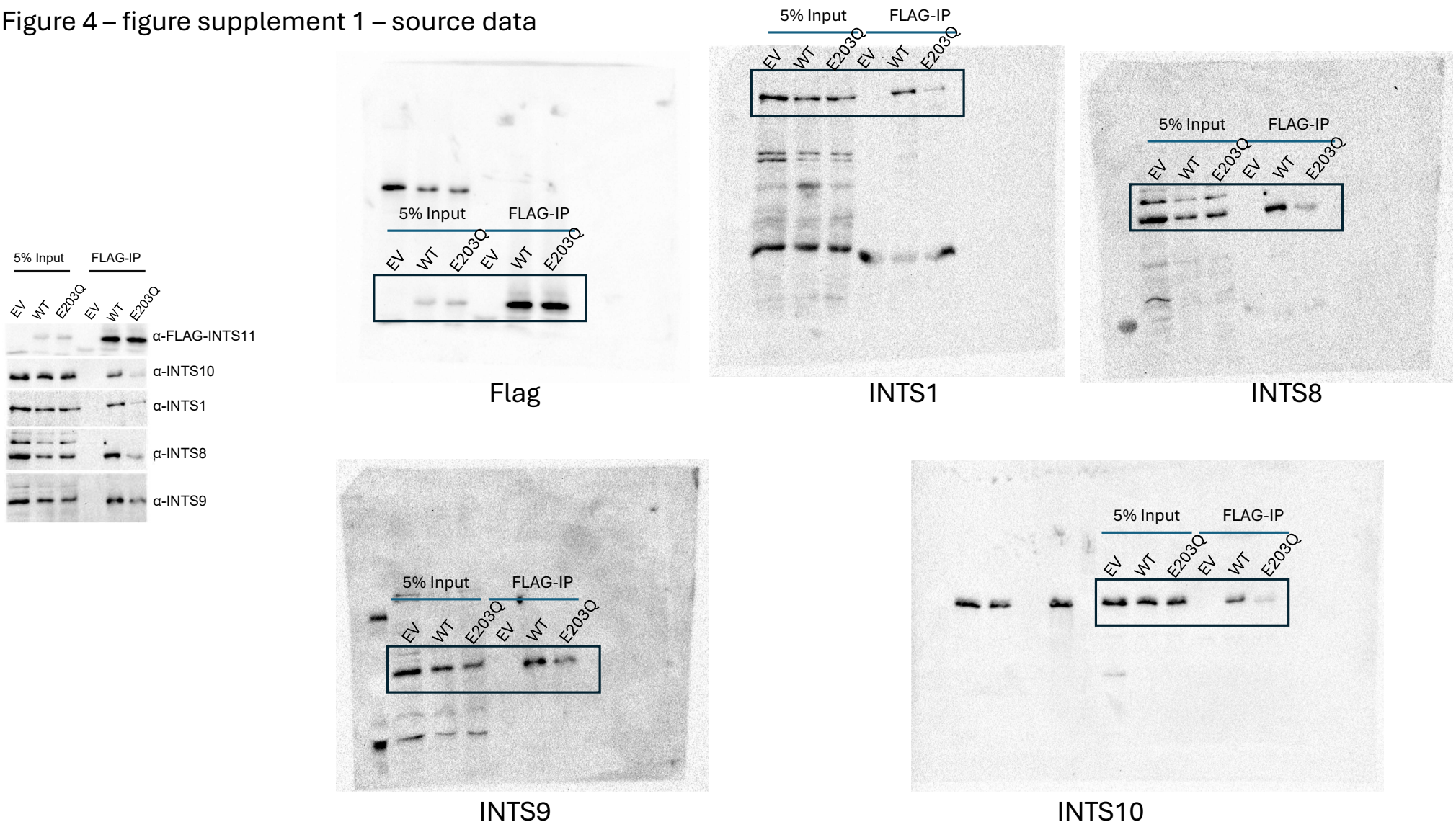

Supplement: Figure 4—figure supplement 1—source data 2. [file elife-92764-fig4-figsupp1-data2.pdf]
